# *In planta* expression of hyperthermophilic enzymes as a strategy for accelerated lignocellulosic digestion

**DOI:** 10.1038/s41598-017-11026-1

**Published:** 2017-09-13

**Authors:** Bilal Ahmad Mir, Alexander A. Myburg, Eshchar Mizrachi, Don A. Cowan

**Affiliations:** 1 Centre for Microbial Ecology and Genomics, Department of Genetics, University of Pretoria, Private Bag X20, Pretoria, 0028 South Africa; 2 Department of Genetics, Forestry and Agricultural Biotechnology Institute (FABI), University of Pretoria, Private Bag X20, Pretoria, 0028 South Africa; 3 Department of Botany, School of Life Sciences, Satellite Campus Kargil, University of Kashmir, Jammu & Kashmir, India

## Abstract

Conversion of lignocellulosic biomass to biofuels and biomaterials suffers from high production costs associated with biomass pretreatment and enzymatic hydrolysis. *In-planta* expression of lignocellulose-digesting enzymes is a promising approach to reduce these cost elements. However, this approach faces a number of challenges, including auto-hydrolysis of developing cell walls, plant growth and yield penalties, low expression levels and the limited stability of expressed enzymes at the high temperatures generally used for biomass processing to release fermentable sugars. To overcome these challenges we expressed codon-optimized recombinant hyperthermophilic endoglucanase (EG) and xylanase (Xyn) genes in *A*. *thaliana*. Transgenic *Arabidopsis* lines expressing EG and Xyn enzymes at high levels without any obvious plant growth or yield penalties were selected for further analysis. The highest enzyme activities were observed in the dry stems of transgenic lines, indicating that the enzymes were not degraded during stem senescence and storage. Biomass from transgenic lines exhibited improved saccharification efficiency relative to WT control plants. We conclude that the expression of hyperthermophilic enzymes in plants is a promising approach for combining pretreatment and enzymatic hydrolysis processes in lignocellulosic digestion. This study provides a valid foundation for further studies involving *in planta* co-expression of core and accessory lignocellulose-digesting enzymes.

## Introduction

Dwindling fossil resources and concerns about greenhouse gas emissions have catalyzed a worldwide interest in the exploitation of lignocellulosic plant biomass, the most abundant renewable and low-cost organic raw material, for production of biofuels and biomaterials^1, 2^. Lignocellulosic biomass is mainly composed of cellulose and hemicellulose, embedded in highly cross-linked lignin polymers which protect the polysaccharides from chemical and enzymatic degradation. The efficient enzymatic conversion of recalcitrant plant cell wall structural biopolymers into fermentable sugars remains a major challenge to the biofuel processing industry due to the high production costs associated of the enzymes required to disrupt the lignocellulosic biomass^3–6^. Production of lignocellulose-digesting enzymes directly within the feedstocks, a promising approach, may provide more cost-effective, and less capital-intensive alternatives than independent microbial fermentation^5–11^, and could reduce the mass transfer limitations of enzyme diffusing into the complex polymeric substrate matrix^6, 12^.

Despite these potential advantages, *in planta* expression of lignocellulose-digesting enzymes from mesophilic bacteria and fungi, typically active at ambient plant growth temperatures, face a number of performance challenges. These include the auto-hydrolysis of developing cell walls, stunted plant stature, yield penalties, poor seed set and germination, reduced fertility and increased susceptibility of the host to disease^13–17^. In addition, the harsh conditions required for pretreatment of lignocellulosic biomass prior to enzymatic saccharification, such as high temperature steam explosion, extreme pH values or strong salt solutions, may completely denature plant-expressed mesophilic enzymes before they can affect significant de-polymerization and saccharification.

*In planta* consolidated bioprocessing using hyperthermophilic (HT) lignocellulose-degrading enzymes is, at least conceptually, a promising strategy for conversion of lignocellulose into fermentable sugars because these enzymes will continue to function during the ‘heat-up’ phase of a steam explosion process used for lignocellulose pretreatment^17^. HT enzymes should be essentially inactive at ambient plant growth temperature, thereby ensuring normal plant growth and development at physiological temperatures^14, 17–19^. Saccharification efficiency of plant polysaccharides by plant expressed and/or exogenous thermophilic biomass-degrading enzymes has been reported to be high because of low resistance from mass transfer, least non-selective binding of lignin and close proximity to the cell wall polymers^12^. Thermophilic enzymes also shorten the incubation time, and may reduce or even eliminate the risk of downstream contamination in contrast to mesophilic enzymes^12, 20^.

To the authors’ knowledge, expression of HT enzymes in plants has been reported in only a few instances^11, 14, 18, 19^ and the functional *in planta* expression of recombinant HT lignocellulose-digesting enzymes and their auto-hydrolysis has not previously been described. However, plant expressed thermophilic enzymes (reviewed in ref. 17) have been reported to significantly increase the efficiency of saccharification compared to addition of exogenous commercial enzymes^12, 21^. The use of enzymes that function optimally under harsh pretreatment conditions opens the way to develop combined pretreatment and enzymatic hydrolysis strategies for the efficient conversion of lignocellulosic plant biomass into fermentable sugars. To investigate the potential benefits of *in planta* expression of lignocellulose-digesting HT enzymes, we here describe the apoplastic expression of recombinant HT endo-1,3-β-glucanase (EG) and β-1,4-xylanase (Xyn) in *Arabidopsis*. The EG is a multi-domain enzyme known to retain crystalline cellulose-hydrolyzing activity at around 100 °C and to be inactive below 50 °C^22^. The Xyn used is a hyperthermostable enzyme with a temperature optimum of 80 °C, stable at alkaline pH and with very limited activity at plant growth temperatures^23^. These enzymes were chosen to be compatible with high temperature and high pH lignocellulose pretreatment processes.

## Results

### Genetic engineering EG and Xyn genes for expression in plants

**Figure 1:**
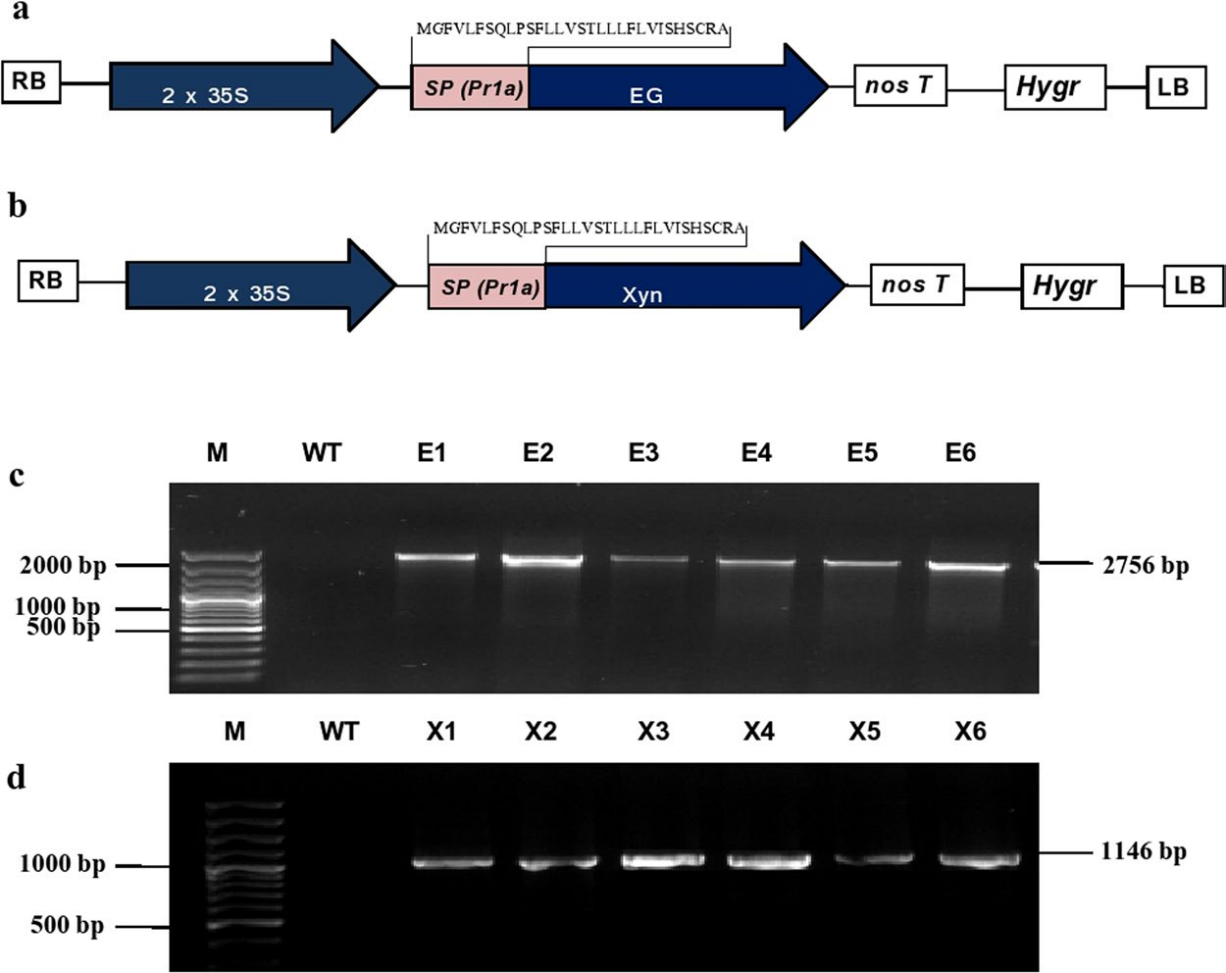
(**a-b**): Schematic representation of EG (**a**) and Xyn (**b**) expression constructs used for constitutive expression in *Arabidopsis* plants using pMDC32; (**c-d**) PCR analysis of genomic DNA from *A. thaliana* plants transformed with EG (**c**) and Xyn (**d**). 2X35S, Cauliflower mosaic virus (CaMV) 35SS promoter; SP, tobacco pathogenesis related protein 1a (Pr1a) signal peptide; NOS, nopaline synthase transcriptional terminator; *Hyg* ^r^, Hygromycin resistance gene as a selection marker; LB and RB, left border and right border respectively. The codon optimized N-terminal amino acid sequence of Pr1a is shown in the construct. E1, E2, E3, E4, E5, and E6 represent EG expressed transgenic lines; X1, X2, X3, X4, X5 and X6 represent Xyn expressed *Arabidopsis* lines. WT represents the wild type control plants, M, DNA marker ladder.

The codon usage of the EG and Xyn genes was modified for expression in *Arabidopsis* and the native signal peptide (SP) of the optimized EG and Xyn genes were replaced by SP of the tobacco pathogenesis-related protein (Pr1a) for cell wall targeting. The codon optimization resulted in a GC content of the EG and Xyn genes of 42% and 43%, respectively, compared to a GC content of the non-optimized EG and Xyn genes of 50% and 61.5%. Both recombinant genes were inserted in between the CaMV35S promoter and Tnos sequence of the plant binary expression/transformation vector, pMDC32 (Fig. 1a,b) to allow constitutive expression of EG and Xyn in *Arabidopsis*. Using the computer programs TargetP and SignalP 3.0^24, 25^, both proteins were predicted to be secreted, with a cleavage site between amino acids 30 and 31 (Supplementary Fig. 1). Glycosylation sites of the recombinant proteins were also predicted using NetNGlyc (Supplementary Fig. 1).

### Expression of EG and Xyn in *Arabidopsis*

**Figure 2:**
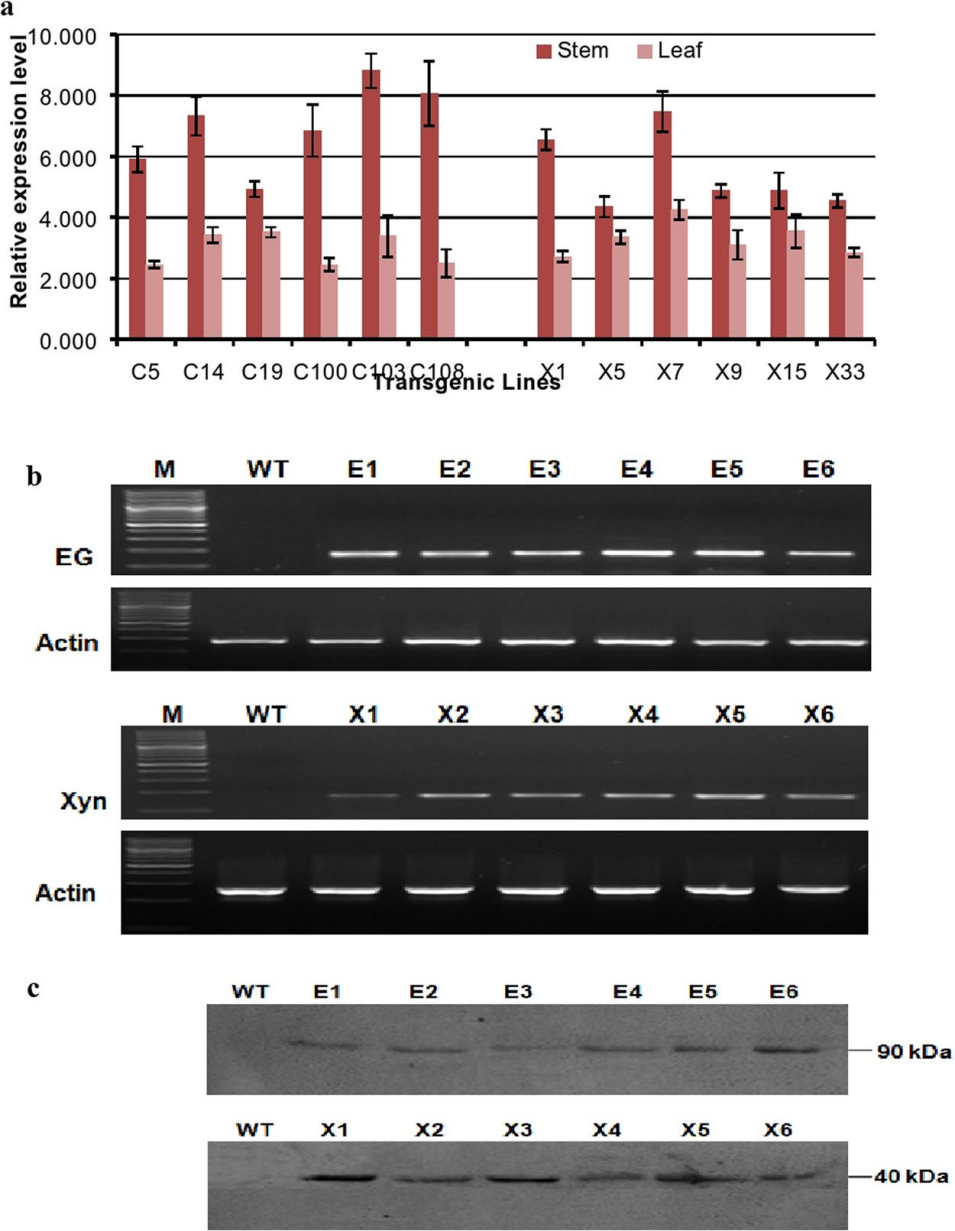
Expression of EG and Xyn in *Arabidopsis*. (**a**) Quantitative RT-PCR analysis of the transgenic lines. EG and Xyn transcripts were measured by RT-qPCR in EG-over-expressed (E1, E2, E3, E4, E5, and E6) and Xyn-over-expressed (X1, X2, X3, X4, X5 and X6) *Arabidopsis* plants; (**b**) EG and Xyl expression in four week old *Arabidopsis* transgenic plants. WT represents the wild type control plants, M, DNA marker ladder. The values are mean ±SD of three biological replicates of each line. Actin was used as an internal control for QRT-PCR analysis; (**c**) Western blot analysis of total soluble proteins (TSP) extracted from the six week old stems of T3 homozygous transgenic *Arabidopsis* plants. Equal amount (30 µg) of TSP loaded to each well were electrophoresed through 12% SDS-PAGE, transferred onto a PVDF membrane and proteins detected by SuperSignal^®^ West Pico (Pierce Biotechnology Inc.; Rockford, IL) Chemiluminescent substrate for the horseradish peroxidase reaction using custom synthesised peptide based rabbit anti-EG and anti-Xyn polyclonal antibodies as the primary antibody and anti-rabbit antibodies conjugated to horseradish peroxidase (Amersham) as the secondary antibody. Protein extracts from non transgenic wild type (WT) stems were used as negative control on both blots.

Positive transgenic (T1) *Arabidopsis* lines containing EG and Xyn were confirmed via genomic PCR using gene specific primers (Fig. 1c,d). No PCR band was observed for untransformed WT *Arabidopsis*. We examined the expression of EG and Xyn in transgenic plants by RT-PCR using RNA isolated from leaves and stems (Fig. 2b). Six independent transgenic lines; E1, E2, E3, E4, E5, and E6 (transformed by pMDC32::EG) and X1, X2, X3, X4, X5 and X6 (transformed by pMDC32::Xyn)showing good expression levels without any phenotypic differences from the WT plants were selected for further analysis. Positively tested T1 lines showing 3:1 segregation, consistent with a single locus insertion, were then used to produce homozygous T3 lines for further analysis. Transcript levels measured by Quantitative RT-PCR showed that the EG and Xyn genes were expressed in transgenic *Arabidopsis*, with expression being higher in stems than in leaves (Fig. 2a). Among the EG lines, line E5 had the highest expression while line E2 showed moderate expression (Fig. 2a). Similarly, line X3 showed the highest transcript abundance among the Xyn expression lines. The expression of EG and Xyn in the stems of T3 homozygous plants was further confirmed by western blot analysis using custom-synthesized peptide-based antibodies (Fig. 2d). Based on the migration of molecular weight markers, EG and Xyn were detected at around 90 kDa and 35 kDA, respectively, which corresponds to the theoretical size of their native sequences after cleavage of the Pr1a signal peptides (Fig. 2d). The EG and Xyn proteins accounted for up to 10.9% and 8.5% of TSP in E5 and X3 transgenic lines, respectively.

**Figure 3:**
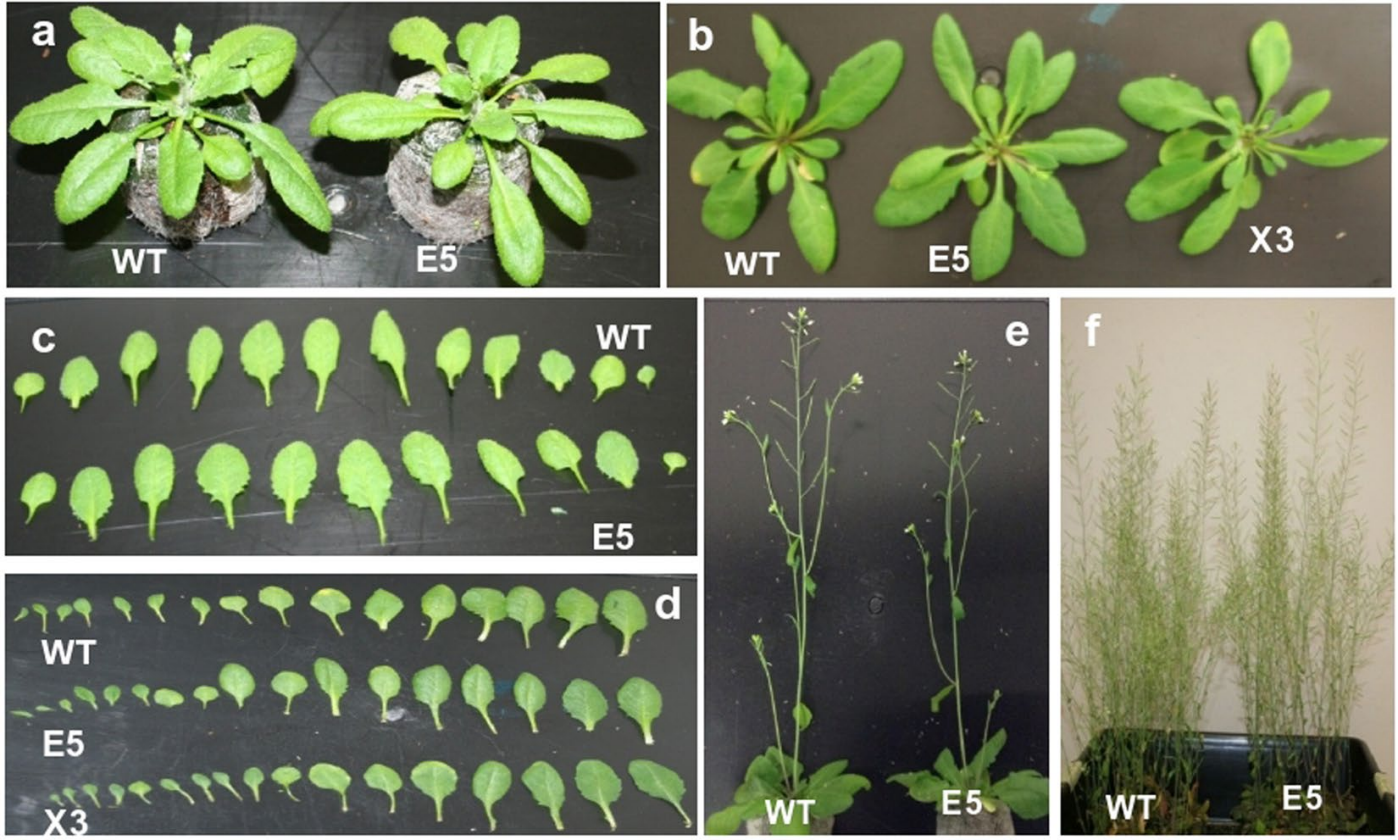
Effect of EG and Xyn heterologous expression in *Arabidopsis*. (**a**) WT and E5 *Arabidopsis* at four-week-old. (**b**) WT, E5 and X3; (**c-d**) Rosette leaves in (**a**) (WT versus E5) and (**b**) (WT versus E6 and X3) from the first to last are arranged from left to right; (**e**) WT and E5 in *Arabidopsis* at 6 weeks old. f) *Arabidopsis* plants at maturity (8 week) bearing siliques and displaying normal growth.

On visual comparisons, the morphology and growth behavior of the homozygous T3 transgenic plants were generally similar to those of WT plants, although some transgenic plants transformed with Xyn exhibited a shorter stature compared to WT plants (Table 1). The plants transformed with EG displayed normal growth and development with regard to plant height and fresh weight (Fig. 3 and Table 1).

**Table 1:** Plant height and fresh weight of seven week old wild type (WT) and homozygous T3 plants of *Arabidopsis*, and total soluble proteins (TSP).

Plant line	Height (cm) Mean ± SD (n = 20)	Fresh weight (g) Mean ± SD (n = 20)	TSP*(%)
WT	**33.3 ± 3.1**	**1.84 ± 0.08**	**—**
E1	31.6 ± 2.6	1.63 ± 0.10	9.85
E2	31.2 ± 1.8	1.65 ± 0.12	10.3
E3	31.5 ± 2.6	1.60 ± 0.08	8.90
E4	32.0 ± 1.9	1.75 ± 0.07	9.3
E5	33.7 ± 1.7	1.78 ± 0.12	10.9
E6	32.8 ± 2.5	1.78 ± 0.06	10.5
**Mean**	**32.13 ± 0.90**	**1.70 ± 0.08**	**9.96**
X1	31.4 ± 1.8	1.64 ± 0.06	8.20
X2	30.0 ± 2.8	1.65 ± 0.07	7.5
X3	32.5 ± 0.9	1.72 ± 0.12	8.5
X4	30.3 ± 2.3	1.55 ± 0.10	7.70
X5	30.6 ± 1.5	1.60 ± 0.09	7.75
X6	29.0 ± 2.6	1.52 ± 0.11	7.45
**Mean**	**30.78 ± 0.95**	**1.61 ± 0.07**	**7.85**

*Total soluble proteins (TSP).

### Biochemical properties of plant-derived HT enzymes

**Figure 4:**
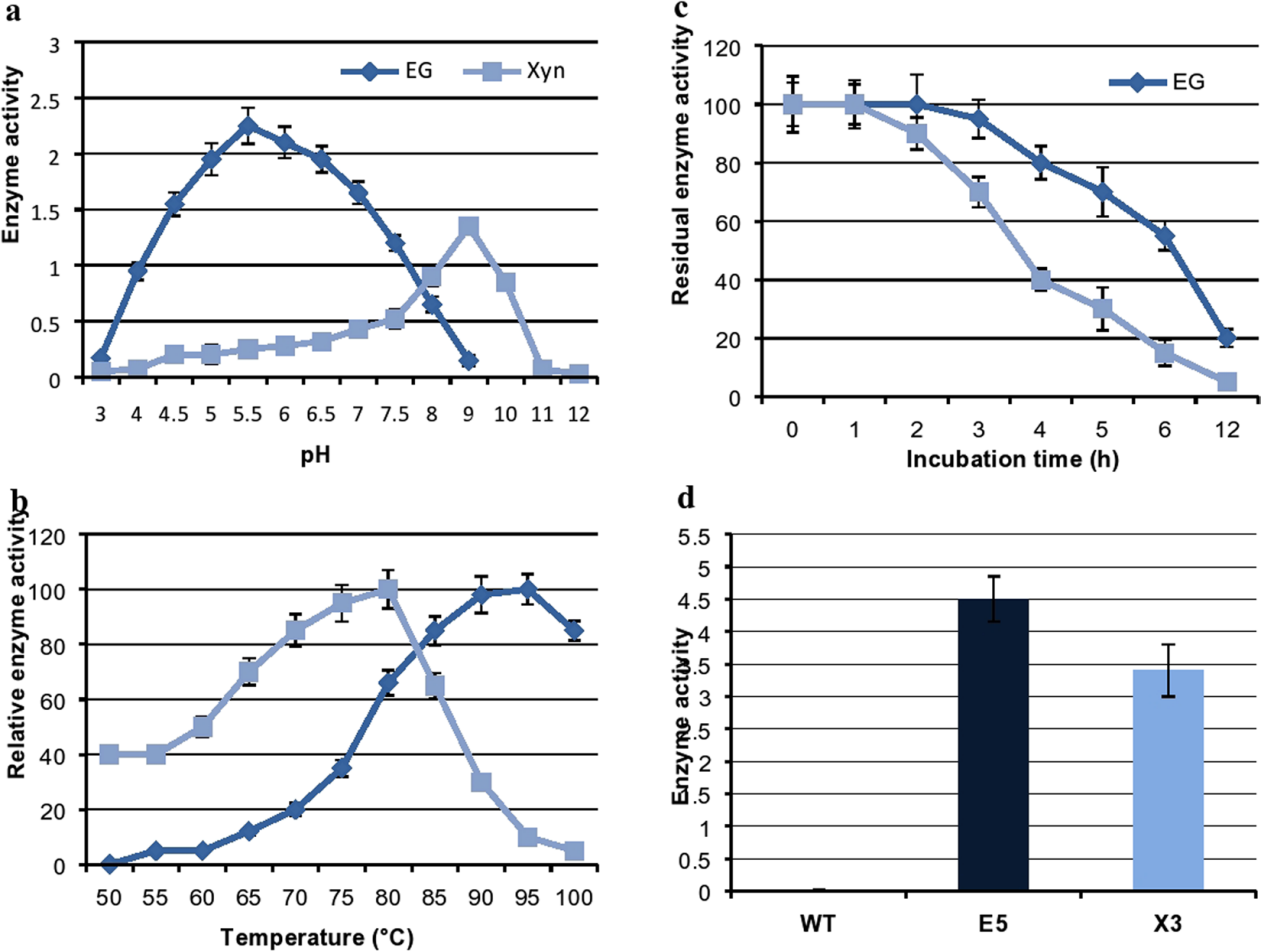
Effect of pH and temperature on the activity and stability of endoglucanase (EG) and xylanase (Xyn). (**a-b**) The recombinant endoglucanase and xylanase incubated in various buffers (pH 3–12) and temperatures (50–100 °C) and assayed for enzyme activities. Measurements shown in (**a**) were performed at 90 °C for EG and 80°C for the Xyn. Measurements shown in (**b**) were obtained using optimal pH for EG and Xyn as shown in A. (**c**) Recombinant enzyme extracts were incubated in buffers of optimum pH without substrates and kept at 90 °C for EG and 80 °C for the Xyn. Aliquots were collected at various time intervals and stored at 0 °C for calculating residual activity. Highest enzyme activity for each enzyme at time point zero is set to 100%. (**d**) Similarly enzyme extracts after incubation at their respective temperature and pH optima along with the WT extracts for 30 min were assayed for enzyme activities. All the measurements were performed in triplicate. The activity is expressed as µmol of reducing sugars min^−1^(U) mg^−1^ protein; for (B & C) enzyme activities are expressed as relative activity percentage where highest enzyme activity is set to 100%. Each data point represents the average enzyme activity from three biological replicates and shown as the average ± standard deviation. Activity was measured in 1% CMC in 50 mM HEPES buffer for endoglucanase and 1% Beachwood xylan in glycine-NaOH buffer for xylanase. All the transgenic lines for EG and Xyl showed similar temperature/pH versus activity profiles. The products were detected by DNS reducing sugar assay using glucose as a standard.

The temperature and pH optima of plant-expressed endoglucanase were determined to be 95 °C and pH 6.0, respectively (Fig. 4a,b). The enzyme retained 80% residual activity at 95–100 °C and actively hydrolyzed CMC over a broad pH range, with over 50% activity between pH 4.5 to 7.5 (Fig. 4a). The endoglucanase retained ~80% residual activity at pH 5.0 and the xylanase retained ~65% residual activity at pH 8.0 (Fig. 4a). Determination of the temperature and pH profiles of the expressed xylanase on beach-wood xylan gave values of 80 °C and pH 9.0, respectively (Fig. 4a,b). The xylanase was also active over a broad pH range, with over 60% activity between pH 8.0 and 10 but little activity below pH 7.0, indicating the alkaliphilic nature of this recombinant enzyme. The enzyme retained around 60% residual activity at 85 °C (Fig. 4b). The major difference in the temperature profiles of the two enzymes was the higher relative activity of the endoglucanase compared to xylanase at higher temperatures. The activity of both recombinant plant-expressed enzymes was greatly reduced at lower temperatures, with little or no activity detectable below 50 °C (Fig. 4b).

The recombinant endoglucanase and xylanase were partially purified from crude plant extracts by thermal precipitation. Heating the protein extracts to 70 °C for 30 min caused more than 90% of the total proteins to precipitate whereas EG and Xyn remained soluble and active. The heat stability of plant-expressed endoglucanase and xylanase was analyzed by incubation of stem extracts at optimal conditions (90 °C, pH 6.0 for endoglucanase) and (80 °C, pH 9.0 for xylanase) for 12 h (Fig. 4c). The half-life (T_1/2_) values of endoglucanase and xylanase were 4 h at 90 °C, pH 6.0 and 2.5 h at 80 °C, pH 9.0, respectively. The residual enzyme activities were determined hourly over the incubation period (Fig. 4c). The enzymes retained 30–75% of initial activities after incubation for several hours, and endoglucanase retained 20% of initial activity even after 12 h incubation at 90 °C (Fig. 4c). After 3 h of incubation, endoglucanase and xylanase showed residual activities of 95% and 70%, respectively, values which are similar to the heat stability of the enzymes when expressed in bacteria^22, 23^. Similarly, WT control and transgenic EG (E5) and Xyn (X3) plants were evaluated for enzyme activity after heat activation for 30 min at 80 °C and observed significantly higher enzyme activities in the transgenic extracts (Fig. 4d).

### Enzymatic activity

**Figure 5:**
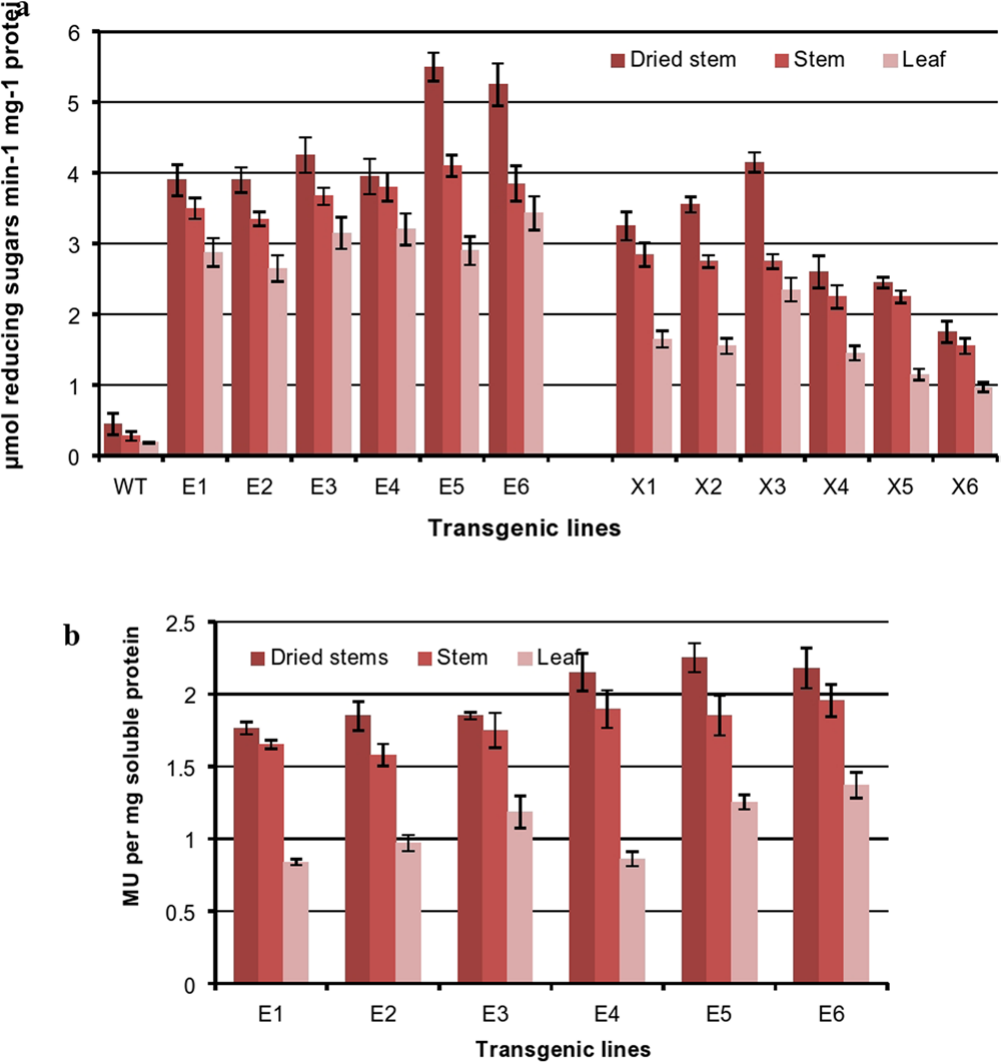
Enzymatic activities in various tissues of transgenic lines. (**a**) Enzyme activity in protein extracts of six transgenic Arabidopsis lines expressing EG (E1, E2, E3, E4, E5 and E6) and Xyn (X1, X2, X3, X4, X5 and X6). Enzyme activity was determined as the release of reducing sugars using glucose as standard on 1% CMC for endoglucanase and 1% beech-wood xylan for xylanase after incubation for 10 min at 95 °C. (**b**) MUCase activity in protein extracts of six western blot positive transgenic *Arabidopsis* lines expressing EG. Activity was expressed as nmol MU mg^−1^ TSP min^−1^. Each data point was determined in triplicate and shown as the average ± standard deviation.

The activity of plant-expressed EG and Xyn proteins from six T3 transgenic lines of each type was determined by measuring the release of reducing sugars from their respective substrates. Endoglucanase and xylanase specific activities among the transgenic lines ranged from 3.9 (E1) to 5.5 (E5) and 1.75 (X6) to 4.15 (X3) µmol reducing sugar/mg protein/min, respectively, in dried extracts of different transgenic lines (Fig. 5a). In contrast to WT plants, which had extremely low endogenous enzyme activities (0.19 to 0.45 µmol reducing sugar/mg protein/min) in all three tissues analyzed, all transgenic lines showed very high enzyme activities (Fig. 5a,b). The activity of the enzymes drops sharply as the temperature declines, displaying limited activity at 50 °C and virtually no activity at temperatures below 30 °C (Fig. 4b), suggesting there would be no hydrolysis of plant lignocellulose under normal plant growth conditions. Enzyme activities in the protein extracts of transgenic stems was also confirmed by 1% CMC and 0.1% RBB-xylan plate assays incubated at 55 °C for 30 min (Supplementary Fig. 3). In all transgenic lines, the lowest activity was found in leaves and the highest in completely dried stems (Fig. 5a). In accordance with the results of QRT-PCR and western immunoblot analyses, transgenic line E5exhibited the highest endoglucanase specific activities (>5 µmol reducing sugar/mg protein/min and 2.2 nmol MU/mg protein/min), while transgenic line X3 yielded the highest xylanase specific activity (4.15 µmol reducing sugar/mg protein/min; Fig. 5a,b). The highest specific activities in stems of E5 and X3 were approximately 11- and 8.3-fold higher than in WT plants. From a comparison of activity data it was clear that EG transformed lines showed higher enzyme specific activities than Xyn transformed lines. Neither stem senescence nor long stem storage of dry stems at room temperature (RT) significantly reduced enzyme activities.

### Enhanced saccharification of transgenic *Arabidopsis* plants

**Figure 6:**
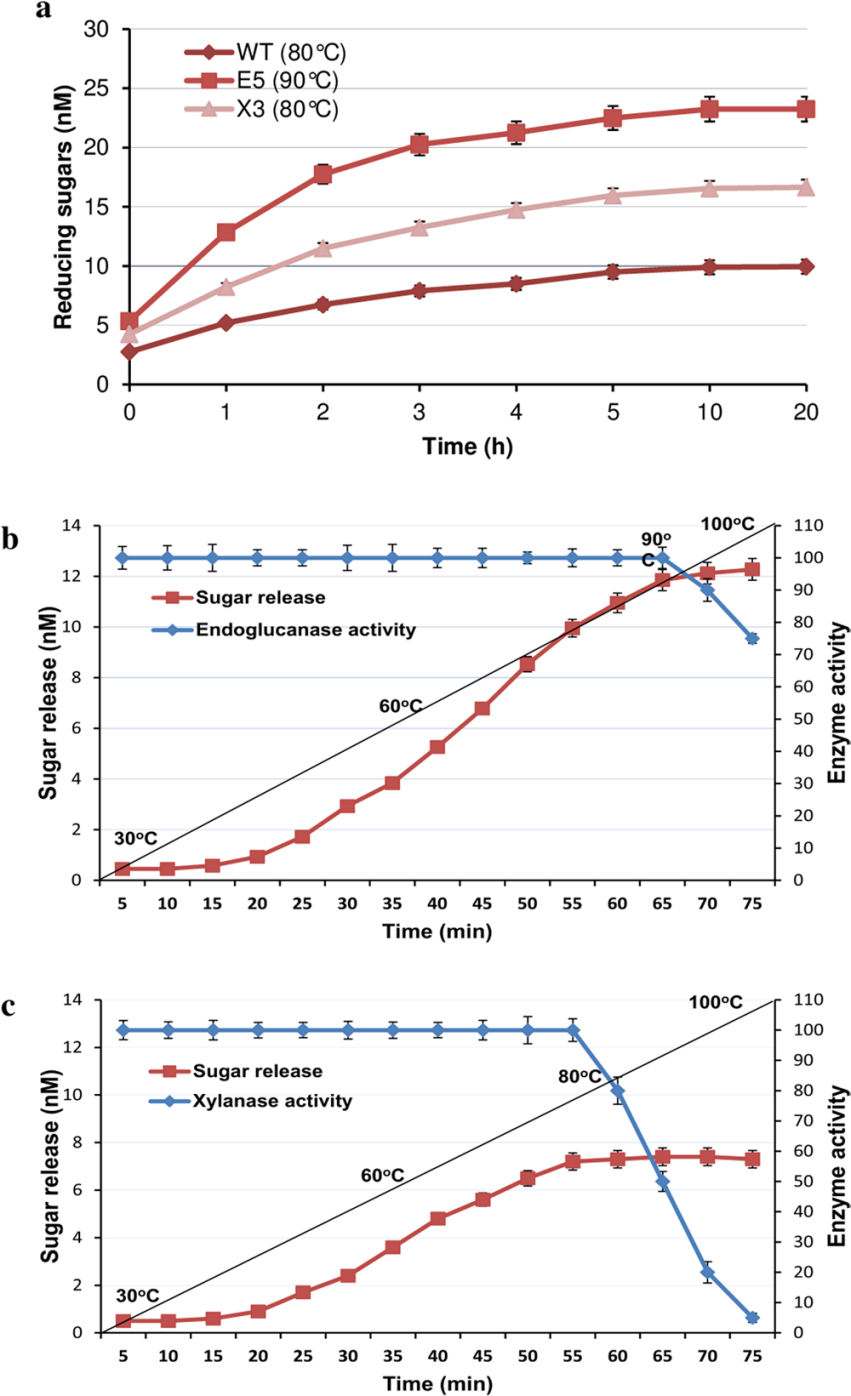
**a**) Saccharification of EG (E5) and Xyn (X3) over-expressing lines of *Arabidopsis* and wild type plants (WT). Dried stem tissues (100 mg each) were ground into powder and incubated for 2 h at RT and then transferred to 80 °C (Xyn) and 90 °C (EG) for 20 h in a 5 ml reaction mixture containing optimum buffers for their activity. Reducing sugars in the hydroysates taken after 1, 2, 3, 4, 5, 10 and 20 h time intervals after centrifuged were measured by DNS assay using glucose as standard. (**b**) E5 and X3 transgenic lines were subjected to saccharification as above for 75 minutes with 1 °C increase in temperature after every 1 minute time period. The lysates taken after every 5 min were assayed for enzyme activity as well as release of sugars. The black line represents the temperature gradient depicting 1 °C increase in temperature after every 1 minute. Activity was expressed in nM of reducing sugars measured after the hydrolysis. The error bars represent standard deviation.

To determine whether plant-expressed HT endoglucanase and xylanase could solubilize *Arabidopsis* lignocellulosic polysaccharides, dried stem extracts of control WT and transformed *Arabidopsis* lines (E5 and X3) were prepared in the buffers of optimum pH strength (50 mM potassium phosphate buffer (pH 6.5) for E5 and 50 mM glycine-NaOH buffer (pH 9.0) for X3) and incubated for few hours at RT. The transgenic lines were then incubated for 20 h at their optimum temperature (90 °C for E5 and 80 °C for X3) for auto-hydrolysis without the addition of external enzymes. WT control plants were incubated under the similar processing conditions for comparison. The aliquots were taken hourly and centrifuged to remove the particulate fraction, and estimation of the soluble sugar content. The kinetic changes in sugar yield from two transgenic biomass expressing endoglucanase or xylanase and WT control follows a typical profile: a rapid initial increase followed by a slow rising phase and a final plateau after 10 h (Fig. 6a). The transgenic lines demonstrated consistently improved hydrolysis compared with the WT control plants through the time course. In addition to the improved sugar yields from both transgenic plants, the results from this hydrolysis also showed a higher initial slope in the sugar yield (Fig. 6a), indicating rapid initial hydrolysis with the transgenic plants than the WT plants. Both endoglucanase-expressing and xylanase-expressing transgenic lines showed two-fold and 1.5-fold improvement in sugar yields than WT biomass, respectively (Fig. 6a), suggesting that plant-expressed enzymes make lignocellulose more accessible and digestible under these processing conditions. It was also noted that hydrolysis of endoglucanase-expressing biomass achieved ~50% improvement than xylanase-expressing transgenic lines (Fig. 6a). This higher release of soluble sugars from endoglucanase transformed biomass may be attributed to the efficient auto-hydrolysis of the crystalline cell wall polysaccharides by the plant expressed endoglucanase as reported previously^22^.

To further explore the effect of temperature and time course on the simultaneous enzyme activity and hydrolysis efficiency an alternative experiment for selected transgenic plants was conducted to ascertain that the improved hydrolysis performance is because of plant-expressed enzymes. The transgenic plants (E5 and X3) were examined for simultaneous enzyme activity and sugar release over a period of 75 minutes without the addition of the external enzyme loadings and both parameters were determined after every 5 min across a temperature gradient of 30–100 °C (Fig. 6b,c). As expected, the initially enzyme activity and sugar release was almost negligible as the temperature was below 40 °C. However, as the temperature increased through the time course kinetic changes in sugar yield from two transgenic plants showed a rapid initial increase from 60 °C to 90 °C (Fig. 6b,c). The activity of the enzymes also increased sharply as the temperature increased up to their optimum temperatures (90 °C for EG and 80 °C for Xyn), suggesting that plant-expressed enzymes increase the hydrolysis and cellulose accessibility. Notably, transgenic EG expressing *Arabidopsis* showed a better enzyme activity with better thermal tolerance and higher sugar yield compared to xylanase transformed line which corresponds to the efficient activity of EG on available cellulose. Further increase in the temperature beyond the optimum temperature of the enzymes did not show significant differences in their sugar yield and the sugar curve showed a plateau afterwards. This may be due feedback inhibition of the product accumulated or enzyme inactivation. The third possible reason may be that the majority of the polymers get hydrolyzed to sugars.

## Discussion

Plants have been widely used for the large-scale production of recombinant cellulases and xylanases^7–17, 26–29^. However, it has been observed that the expression of mesophilic biomass-degrading enzymes may negatively affect plant growth and development^15, 18, 26^. The harsh biomass pretreatment conditions used prior to enzymatic saccharification (such as steam explosion^30–32^) are generally not compatible with the functional limits of mesophilic enzymes^11^. An alternative strategy which resulted in a high *in planta* yield of enzyme expression without affecting growth or development, and where the expressed enzymes were capable of significant catalytic activity under lignocellulosic pretreatment conditions would represent a substantial technological advance. *In planta* expression of hyperthermophilic lignocellulose-digesting enzymes, which are almost completely inactive under physiological conditions, should ensure normal plant growth and development^7, 11, 14, 18, 19, 33^. The high ‘temperature optima’ and thermostability of these enzymes^34^ should ensure that they remain active for a significant period under conventional steam-explosion pretreatment conditions^17^. The production HT biomass digesting enzymes directly in the biomass targeted for conversion would be more economically and environmentally sustainable than microbial fermentation.

In this study, we have demonstrated the technical feasibility of this novel approach. Recombinant endoglucanase and xylanase genes, derived from hyperthermophilic genome sequence databases, were stably integrated into the *Arabidopsis* genome. Both genes were successfully expressed and translated into active proteins at high levels *in planta*, with a much higher enzyme activities obtained than reported in previous studies^7, 8, 14, 18, 19, 27, 28, 33^. The incorporation of Pr1a sequence (MGFVLFSQLPSFLLVSTLLLFLVISHSCRA) in both genes was designed to target the functionally expressed proteins to the plant apoplast. While we have not demonstrated apoplastic localization experimentally, previous studies have demonstrated that the apoplast can serve as a storage site for larger quantities of functional foreign proteins than the plant cytosol^7, 15, 16, 26–28, 35, 36^. High levels of endoglucanase and xylanase gene transcripts were detected in both leaf and stem tissues, with the highest transcript abundance in the stems of two *Arabidopsis* lines (lines E5 and X3: Fig. 2a). The successful translation of both genes in six independent transgenic lines of each transformation was also confirmed by western blot analysis using custom synthesized peptide-based antibodies.

Phenotype analysis of wild-type *Arabidopsis* plants and transgenic lines expressing either endoglucanase or xylanase genes showed no significant changes in growth parameters (Fig. 3 and Table 1), from which we conclude that the expression of the hyperthermophilic enzyme genes did not negatively affect cell wall integrity. We attribute this to the low catalytic activity of the hyperthermophilic enzymes at plant growth temperatures, confirmed by determination of temperature-activity profiles in plant tissue extracts (Figs 4b and 6b,c) where endoglucanase activity was undetectable below 50 °C. This sharp decrease in the activity of the enzymes as the temperature declines and virtually no activity at temperatures below 30 °C (Figs 4b and 6b,c) suggests that there would be no hydrolysis of plant lignocellulose under normal plant growth conditions. The extent to which the protection of the plant cell wall from degradation is also a function of lack of direct access of apoplastic enzymes to the crystalline cellulose embedded in a matrix of lignin and hemicellulose^37^ is not clear. Nevertheless, we have demonstrated unequivocally that high level expression of lignocellulose-degrading enzymes in plant tissues is feasible.

Enzymatic assays of plant tissue extracts demonstrated that the recombinant hyperthermophilic enzymes showed functional properties which were entirely consistent with their hyperthermophilic origins (Fig. 4) and broadly similar to published data of the recombinant enzymes produced in bacterial hosts^22, 23^. The plant-expressed enzyme demonstrated consistently improved hydrolysis of CMC and beech-wood xylan to fermentable sugars compared to WT control plants (negligible activity)after heat- activation of the extracts under similar processing conditions (Fig. 4d). This study demonstrates the successful production of inactive HT endoglucanase and xylanase at normal growth conditions followed by the induction of enzyme activity at higher temperatures. Interestingly, both enzymes retained their near-native thermostability despite the different post-translational machineries operating in bacteria and plants. We also noted that heterologous proteins remained fully active in plant tissues after drying. This observation has implications for the future practical application of this technology, since biomass intended for saccharification and fermentative processing would typically be transported and stored in a dry or semi-dry state.

High-temperature endoglucanase and xylanase activities were detected in both leaf and stem tissues of all six transgenic lines of each type. In the transgenic *Arabidopsis* lines with the highest expression level, the recombinant endoglucanase and xylanase proteins accounted for 10.6% and 8.5% of total soluble protein, respectively (Table 1). These lines showed significantly higher endoglucanase and xylanase titers than *in planta* expression studies targeting different organelles (vacuoles, apoplast, chloroplast, endoplasmic reticulum^7–12, 14, 18, 19, 21, 33, 38^.

The very high recombinant protein yields observed in this study are likely to be due to a range of factors, including the codon optimization of the genes. We believe that the absence of any significant impairment to the growth and development of the transgenic plants may also be a key factor in generating high expression yields.

Biomass from various transgenic lines was tested for autocatalytic saccharification at elevated temperatures (Fig. 6a–c). Short-term incubations at elevated temperatures (80 °C and 90 °C) gave statistically significant increases in reducing sugar yields compared with high-temperature treated WT *Arabidopsis* tissue (Fig. 6a), which we attribute directly to the action of the catalytically-active hyperthermophilic enzymes. In addition to better hydrolysis, the transgenic *Arabidopsis* expressing EG and Xyn also show more rapid initial hydrolysis than does the control plant, however, hydrolysis of EG transformed plants achieved significantly higher sugar yields than the Xyn transformed ones (Fig. 6a). This is consistent with the observation that transgenic plants as expected showed negligible enzyme activity and sugar release below 50 °C, however as the temperature increased up to their optimum values (80 °C for Xyn and 90 °C for EG) a rapid increase in the enzyme activities and sugar yield was observed (Fig. 6b,c **)**. Under these processing conditions plant expressed EG and Xyn make cellulose more accessible and digestible and show rapid hydrolysis performance. Enzymes accumulated within the plant after activation during processing can commence catalysis immediately *in situ* without the need for transport and diffusion. The efficiency of plant-expressed enzymes is therefore expected to be high because of low resistance from mass transfer and an expected decrease in non-selective binding of the enzymes to lignin or other non-target molecules. Further, the expressed enzymes might be in a proximity to the cell wall polymers, which may directly or indirectly facilitate the hydrolysis.

Given that these two digestion trials were each dependent on the action of a single recombinant enzyme, and given the well-established synergistic effects of multiple catalytic activities (particularly endoglucanase plus xylanases^12^), we would predict that the co-expression of both enzymes in the same tissue would give substantially greater saccharification yields. This approach also offers considerable opportunities for using *in planta* enzyme co-expression as a test-bed for the quantitative assessment of the synergistic benefits of ‘accessory’ enzymes (such as feruroyl esterases, acetyl-xylan esterases, xylosidases, mannanases, arabinosidases, glucuronidases, arabinofuranosidases and other enzymes^39, 40^).

## Conclusion

In conclusion, we have demonstrated that *in planta* expressed enzymes stimulate the saccharification of transgenic biomass under conditions that are compatible with biomass pre-treatment technologies. To our knowledge, EG and Xyn are the first enzymes expressed *in planta* that remain active at 80 °C and 95 °C for few hours. These studies provide the ‘proof-of-concept’: the extent to which these hyperthermophilic enzymes remain active and the ‘integrated’ activity under industrial steam-explosion temperature profiles^17–19^ remains to be assessed. Nevertheless, we argue that a degree of tissue auto-hydrolysis during the early stages of the steam explosion process may have substantial benefits for process parameters (and economics); potentially reducing upper temperature maxima and process durations and the accompanying deleterious effects of high temperature processing (Maillard reactions, oxidation, furfural formation)^12, 41^. *In planta* auto-hydrolysis is unlikely to obviate the need for post-processing enzyme addition (whether the addition of exogenous enzymes or the use of a consolidated bioprocess fermentation)^12, 42^ but could have positive economic effects through a reduction in exogenous enzyme costs or a positive change in the digestion kinetics during a consolidated bioprocess^19, 43^.

This study has been restricted to a model plant species (*Arabidopsis*), and an obvious extension of this study is the use of a dedicated bioenergy feedstock such as *Populus* to ascertain whether saccharification enzymes produced *in planta* have similar effects in plant tissues of different composition (different cellulose/hemicellulose/lignin ratios) and particularly in woody tissues. There is also considerable scope for the targeting of these enzymes simultaneously to different cellular compartments so as to enhance both enzyme expression yields and the efficacy of tissue auto-hydrolysis.

## Methods

### Plant material and growth conditions

Columbia-0 (Col-0) ecotype of *Arabidopsis* was used for this study. For growth of *Arabidopsis* on plates the seeds were surface sterilized and plated onto 1x Murashige and Skoog^44^ media with sucrose (0.5% w/v), agar (0.8% w/v) and pH 5.0. Seeds were incubated in a growth chamber for two weeks at 22 °C with constant light following two days of stratification at 4 °C. Seedlings were grown in peat moss bags (Jiffy Products International AS, Norway) at 23 °C under long day conditions (16 h light, 8 h dark) with a light intensity of 120–130 µmol m^−2^ s^−1^.

### Gene synthesis, cloning and transformation

The genes encoding HT endoglucanase, EG (GENBANK accession number JF509452) and xylanase, Xyn (GENBANK accession number AFP81696) selected for this study were codon optimized for expression in *Arabidopsis* and designed such that the 5′ end encoded the tobacco pathogenesis-related protein 1a (Pr1a) signal peptide (MGFVLFSQLPSFLLVSTLLLFLVISHSCRA) for targeting to cell wall. The recombinant genes were synthesized *de novo* by GenScript Corporation (Piscataway, NJ, USA). EG and Xyn genes were amplified (Supplementary Fig. 2) by Phusion DNA Polymerase (New England Biolabs) using gene specific primers (Supplementary Table 1). The PCR products (EG = 2558 bp and Xyn = 1046 bp) were purified by gel extraction (Macherey–Nagel) following agarose gel electrophoresis and used for Gateway BP reaction (Invitrogen). A sequence-verified entry clone was LR recombined with the plant binary vector, pMDC32, to generate the pMDC32::EG and pMDC32::Xyn expression vectors (Fig. 1a,b). The expression of EG and Xyn was controlled by 35SS constitutive cauliflower mosaic virus (CaMV) promoter.

### Genetic transformation and production of transgenic plants

The expression plasmids containing correct coding sequences were transformed into *Agrobacterium tumefaciens* strain LBA4404.*A*. *tumefaciens* (strain LBA4404) containing the desired vectors were grown for 48 hours (28 °C, shake flasks) in LB media with rifampicin (30 mg/L), streptomycin (30 mg/L), and kanamycin (50 mg/L), and used for floral dip transformation as previously described^45^. Transformants were selected by growing T1seeds on 1x MS agar plates (described above) supplemented with 20 μg/ml Hygromycin. Resistant plants were picked after two weeks of growthunder the long day conditions and moved topeat moss bags to raise the transgenic *Arabidopsis* plants for further molecular characterization and enzymatic studies.

### PCR analysis and plant growth studies of transformants

Transgenic *Arabidopsis* plants that constitutively expressed EG and Xyn were generated. Plant lines were separately transformed and regenerated. More than 100 positive transformants from two independent transformation events were selected for each construct based on resistance to Hygromycin. Genomic DNA was isolated from leaves of T1 transgenic plants using the Plant genomic DNA extraction kit (Macherey-Nagel) following the manufacturer’s instructions. Isolated DNA was subjected to PCR to amplify the EG and Xyn genes using primers specific to each (Supplementary Table 1). Agarose gel electrophoresis was used to visualize the amplified products.

Based on PCR genotyping, 50 transgenic plants from two different transgenic events for each construct were evaluated for plant height and fresh weight. The best six T1 lines from each construct were then selected and were self-pollinated to generate a T3 generation for further analysis. Data from T3 transgenic plants (*n* = 20) were analyzed statistically to determine average plant height and fresh weight data.

### Gene expression (RT-PCR and qRT-PCR) analysis

For RT-PCR and qRT-PCR analysis, total RNA was extracted from two-week old *Arabidopsis* leaves and stems using Plant RNA kit (Macherey–Nagel) following the manufacturer’s instructions. RNA (2 µg) was used for cDNA synthesis by incubating the RNA for 30 min at 37 °C in a reaction mix containing 1 µl of RQ1 RNase-Free DNAse (1 U µl^−1^; Promega), 1 µl of 10X RQ1 DNAse buffer (Promega), and RNase-free water to a final volume of 10 µl. The reaction was stopped by adding 1 µl of RQ1 DNase STOP Solution (Promega) and incubating the samples for 10 min at 65 °C to inactivate DNase. For retro-transcription, 2.5 µl of each DNase-treated RNA sample was added to a reaction mix containing 7.0 µl of RNase-free water, 1 µl (500 ng) of random primers (Promega) and 0.5 µl of RNAse inhibitor. After 10 min of incubation at 70 °C, the reaction mixture was cooled on ice. To this reaction mixture was added 4 µl of ImProm-II Reaction Buffer 5X (Promega), 2.4 µl of 25 mM MgCl_2_, 1.6 µl of 2.5 mMdNTP mix and 1 µl of Improm-II RT (Promega). Samples were incubated for 10 min at room temperature (RT, 25 °C), 1 h at 42 °C and 15 min at 70 °C. The cDNA product was then subjected to semi-quantitative PCR using primers specific for the genes of interest (Supplementary Table 1). Synthesised cDNA was further used for qRT-PCR analysis using SYBR Green Master Mix (Roche) in a QuantStudio 12 K Flex (Applied Biosystems). EG and Xyn expression levels in each sample were normalized using Actin and Ubiquitin as internal controls. See Table S1 for primer sequences.

### Protein extraction

Different plant tissues (leaves, green and dried primary stems) were ground in liquid nitrogen to a fine powder using a mortar and pestle, and re-suspended in protein extraction buffer containing 50 mM sodium phosphate (pH 6.5), 0.5 mM NaCl and 2 mM PMSF at 4 °C. The extracts were kept on ice for 20 min and vortexed occasionally during this period. The homogenate was centrifuged at 14000 *g* for 30 min at 4 °C and the supernatants were transferred to new tubes and stored at −20 °C. Bulk proteins were removed by heat precipitation at 90 °C (EG transformed plant tissues) and 80 °C (Xyn transformed plant tissues) for 30 min and after centrifugation at 14000 *g* for 20 min at 4 °C, the supernatants were retained and stored at −20 °C. Total soluble protein (TSP) in the supernatants was measured using the Bradford assay^46^ with bovine serum albumin (BSA, Sigma) as a protein standard.

### Western blot analysis

For western blot analysis, TSP extracted from the six week old stem tissues of non-transgenic and T3 transgenic *A*. *thaliana* plants expressing EG and Xyn were used. TSPs (30 µg) from each sample were electrophoresed in 12% SDS-PAGE gels. After separation, proteins were transferred onto a PVDF membrane (Life Technologies) using aniBOLT 2 gel transfer device (Life Technologies). The membrane was blocked for 1 h with blocking buffer (1x Tris-buffered saline (TBS), 5% non-fat dry milk) at room temperature (RT). The membrane was washed once for 10 min and twice for 5 min with a washing solution (1x TBS, 0.005% Tween-20), then incubated overnight at room temperature in 20 ml of washing solution containing custom synthesized primary antibody against EG and Xyn (rabbit anti-EG and anti-Xyn polyclonal antibodies) at a dilution of 1:1500. The peptide antigens used to raise the polyclonal antibodies were CDLPDKDGKSDGSAN (for EG) and CEGYQSSGSSDITVG (for Xyn). The membrane was washed again as described above and probed with anti-rabbit secondary antibodies conjugated to horseradish peroxidase (Amersham) at a dilution of 1:10000 for another 2 h. Finally, the membrane was washed in washing buffer for 30 min, followed by incubation in Super-Signal^®^ West Pico Chemiluminescent substrate (Pierce Biotechnology Inc.; Rockford, IL) for the horseradish peroxidase (HRP) reaction for 5 min. The membrane was exposed to the X-ray film (CL-xPosure^TM^ Film, Thermo Scientific) and the film was developed in a Kodak RP X-OMAT processor.

### Enzymatic assay of recombinant enzymes

Endoglucanase and xylanase activities were determined quantitatively using the DNSA method. Reaction with 0.95 ml 1% carboxymethyl cellulose (CMC, Sigma) in 50 mM potassium phosphate buffer (pH 6.5) at 90°Cand 1% Beachwood xylan (Sigma) in 50 mM glycine-NaOH buffer (pH 9.0) at 80 °C in temperature controlled water-bath were initiated with 50 µg (1 mg/ml) protein and terminated by the addition of equal volumes of DNS reagent. Reaction mixtures were then boiled for 10 min and A_540_ measured to calculate the reducing sugar, using glucose as a standard. All the assays were performed in triplicates. One unit of enzyme activity is defined as the amount of enzyme that released 1µmol of reducing sugar (glucose) per minute under the assay conditions. Enzyme activities were also visualized on agar plates containing 1% CMC or 1% RBB-xylan (Sigma). Extracts (20 µl) placed on the agar/CMC or agar/RBB-xylan plates were incubating for 60 min at 90 °C and 80 °C, respectively. The plates were stained in 0.1% Congo red solution for 20 min at RT and washed with 1 M NaCl to visualize the zone clearance.

Where indicated, endoglucanase activity was determined using the 4-methylumbelliferyl-β-d-cellobiose (MUC, Sigma) assay^34^. To a 100 µl reaction mixture (50 mM sodium acetate, 100 mM NaCl, 0.5 mM MUC), 50 µg of protein was added and incubated for 30 min at 90 °C. The reaction was stopped by adding 100 μl of 150 mM glycine (pH 10). The amount of released MU was measured with a fluorescence spectrophotometer at excitation and emission wavelengths of 360 and 465 nm, respectively. Endoglucanase activity in each transgenic plant was calculated after subtracting the background activity contributed by the WT non-transgenic control plants. Results were calibrated to standard solutions of MU. Values represent the average of three independent assays using clones of the same transgenic and untransformed lines.

### Biochemical characterization of recombinant enzymes

The stabilities of plant expressed recombinant endoglucanase and xylanase were determined by incubating the protein extracts in 1% (w/v) CMC and beech-wood xylan, respectively prepared in various buffers ranging from pH 3.0 to 12.0. The different buffers used were citrate (pH 3.0 to 6.0), MES (pH 6.0 to 7.0), HEPES (pH 7.5 to 9.0), phosphate buffer (pH 7.0 to 8.0) and glycine-NaOH (pH 9.0 to 12.0). The buffers were used at buffer strength of 50 mM and incubated for 30 min in each buffer. The optimum temperature for the endoglucanase and xylanase activities was determined by conducting enzyme assays over a range of temperatures form 50 to 100 °C at optimum pH with appropriate controls. To determine enzyme thermostability, residual endoglucanase and xylanase activities were measured after incubating protein extracts at 90 °C and 80 °C with the removal of aliquots at regular time intervals and assayed as described above.

### Tissue saccharification

For saccharification experiments, mature stems (500 mg, fresh weight) of non-transformed WT and transformed *Arabidopsis* lines were harvested and dried as described previously^12^. The entire stems were ground to a fine powder and incubated at RT for few hours in the buffers of their optimum pH strength; 50 mM HEPES buffer (pH 6.5) for endoglucanase transformed stems and 50 mM glycine-NaOH buffer (pH 9.0) for xylanase transformed stems. Initially, different buffers were used however the HEPES and Glycine-NaOH buffers gave the best results. Subsequently, tissues were placed for 20 h at 80 °C (Xyn transformed biomass) and 90 °C (EG transformed biomass) for biomass hydrolysis evaluation along with control WT biomass. The hydrolysate aliquots were taken hourly (after 1, 2, 3, 4, 5, 10 and 20 hours) and centrifuged to remove the particulate fraction, and estimation of the soluble sugar content using DNSA with D-glucose as the standard. In parallel experiment, transgenic biomass was again subjected to enzymatic hydrolysis by incubating the biomass for a period of 75 min. To evaluate the effect of plant-expressed HT enzymes on the biomass hydrolysis, enzyme activity and sugar release were determined from the extracts after every 5 minutes across the temperature gradient (from 30 °C to 100 °C) as described above. Each experiment was repeated three times.

## Electronic supplementary material

supplementary information
