# Supplementary material for: In planta expression of hyperthermophilic enzymes as a strategy for accelerated lignocellulosic digestion
Source: Sci Rep. 2017 Sep 13;7:11462. doi: 10.1038/s41598-017-11026-1 (PMC5597601; doi:10.1038/s41598-017-11026-1)
Supplement: Supplementary file 1 — supplementary information [file 41598_2017_11026_MOESM1_ESM.pdf]

***In planta* expression of hyperthermophilic enzymes as a strategy for accelerated lignocellulosic digestion**

**Bilal Ahmad Mir[1,2†], Alexander A Myburg[2], Eshchar Mizrachi[2], Don A Cowan[1‡]**

[1]Centre for Microbial Ecology and Genomics, Department of Genetics, University of Pretoria, Private Bag X20, Pretoria 0028, South Africa

[2]Department of Genetics, Forestry and Agricultural Biotechnology Institute (FABI), University of Pretoria, Private Bag X20, Pretoria 0028, South Africa

[†]Current address: Department of Botany, School of Life Sciences, University of Kashmir, Kargil Campus, Kargil, Jammu & Kashmir, India

[‡]Corresponding author: don.cowan@up.ac.za

**S1:** Prediction of cleavage and N-glycosylation sites of recombinant endoglucanase (a) and recombinant xylanase (b). Sub-cellular localization of the recombinant proteins was predicted using TargetP (Emanuelsson et al., 2000, 2007) and N-glycosylation sites were predicted using NetNGlyc (http://www.cbs.dtu.dk/services/NetNGlyc/).

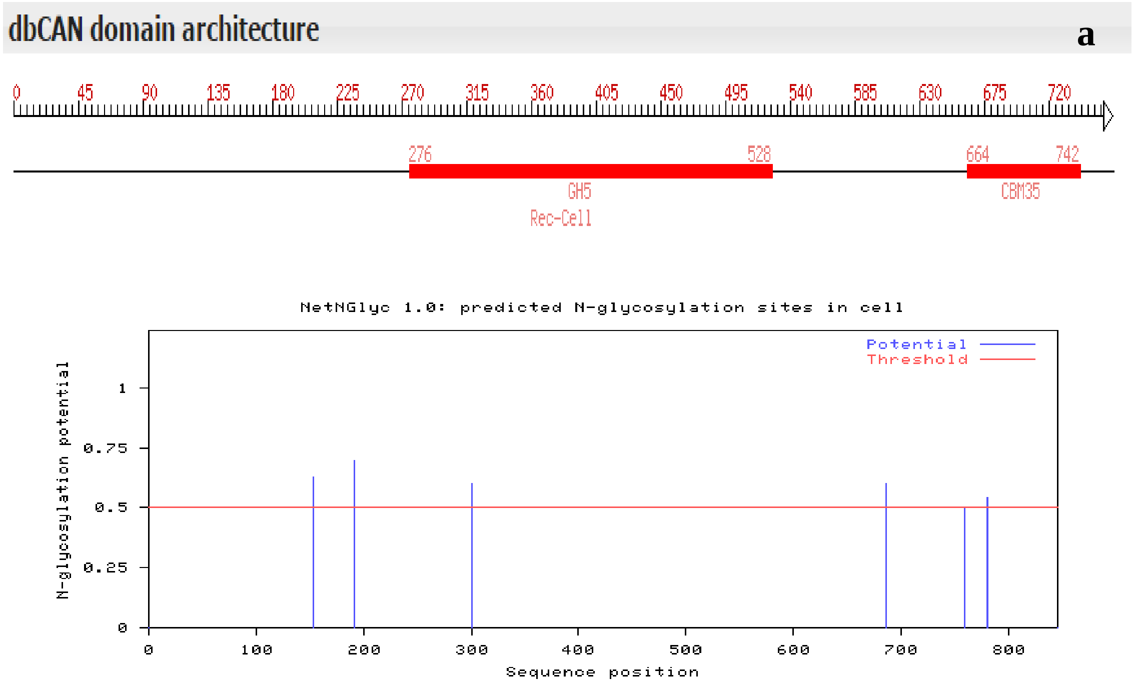

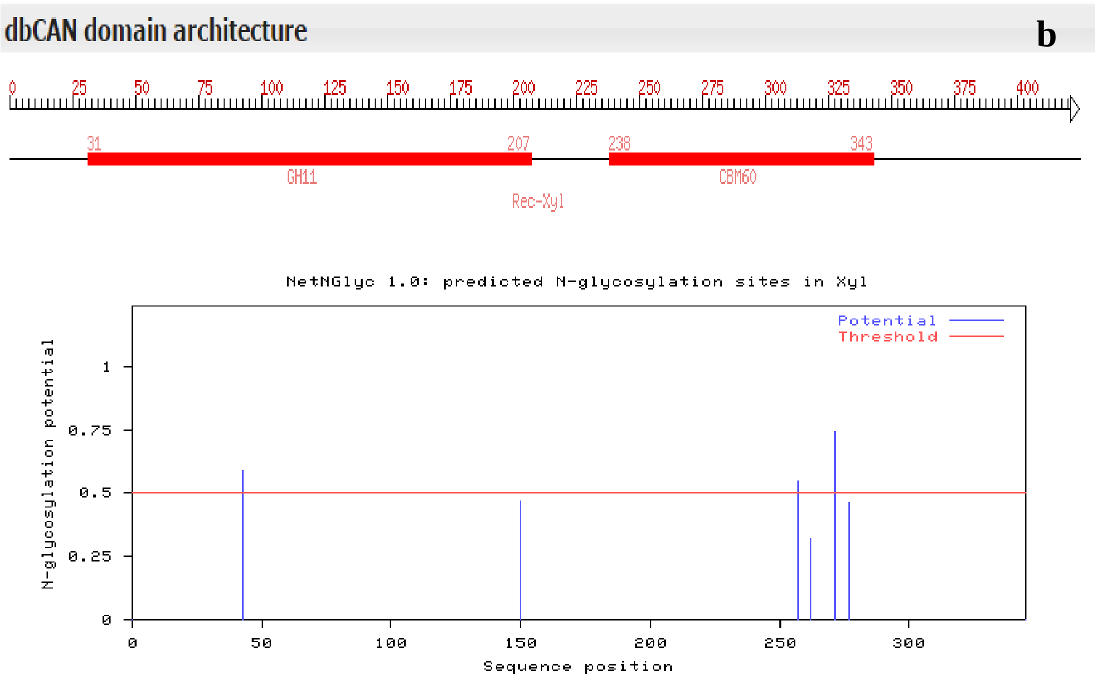


**S2:** Gene cloning of Rec-EG (2756bp), and Rec-Xyn (1146bp) using gene specific primers. M= marker ladders. PCR for EG gene was performed with the following conditions: 30s at 98°C; 30 cycles as follows: 10 s at 98°C; 40 s at 60°C; 1 min at 72°C; 5 min at 72°C. PCR for Xyn was performed with the following conditions: 30s at 98°C; 30 cycles as follows: 10 s at 98°C; 30 s at 60°C; 40s at 72°C; 5 min at 72°C.

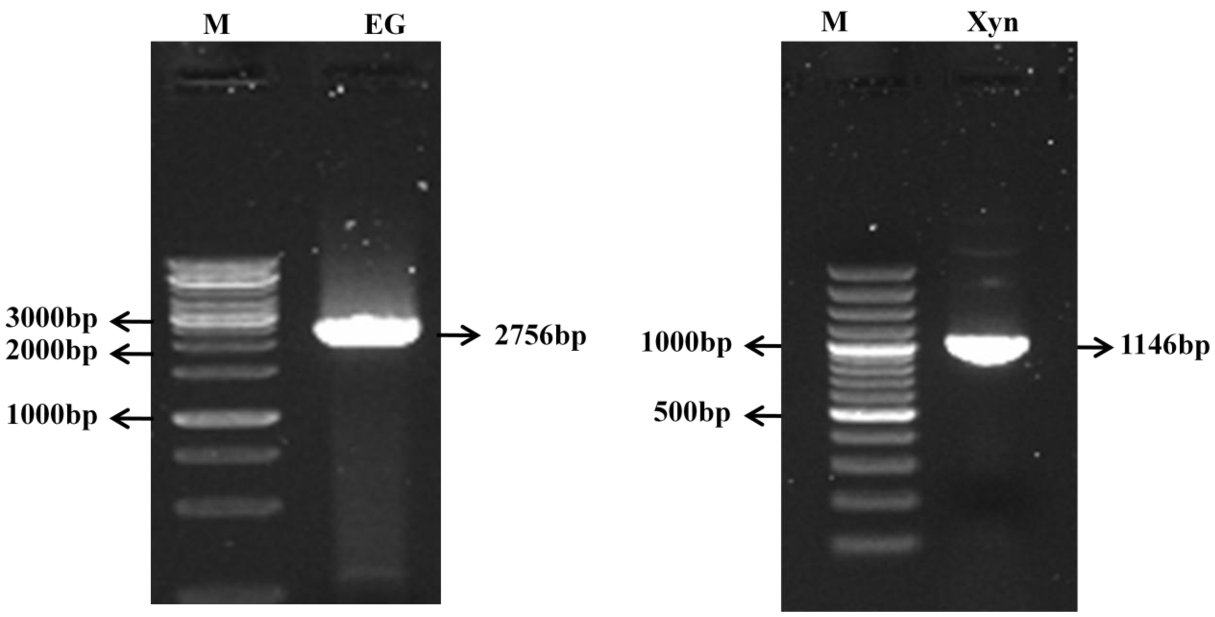

**S3:** Endoglucanase and xylanase activity of protein extracts from C108 and X7 plants expressing Rec-ebi244Opt and Rec-rMxylOpt respectively. A) Observation of clear zone around the protein extracts on 0.1% RBB-xylan (Sigma) plate using 0.1% Congo Red. B-C) Observation of clear zone around the protein extracts on 1% CMC (Sigma) plates using 0.1% Congo Red. The plates were incubated at 75°C for 30 min.

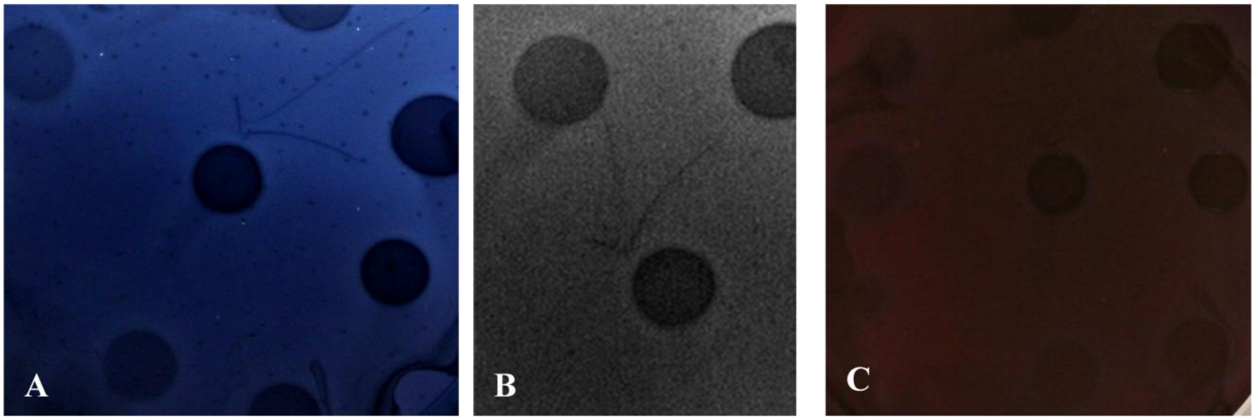

**Table 1: Primers used in this work**

| Primer name | Sequence (5'-3') |
|---|---|
| EGFW1 | CAGCTGGCCACCATGGGATTTGTTCTCTT |
| EGRev1 | CAGCTGTTAGCCAGCCCTAGTGATTTCG |
| XynFW1 | CAGCTGGCCACCATGGGATTTGTTC |
| XynRev1 | CAGCTGTTACGGCGTGTTTCCGTAG |
| EGFW2 | CAGCTGGCCACCATGGGATTTGTT |
| EGRev2 | CAGCTGTTAGCCAGCCCTAGTGAT |
| XynFW2 | CAGCTGGCCACCATGGGATTTGTT |
| XynRev2 | CAGCTGTTACGGCGTGTTTCCGTA |
| EGFW3 | CAGCTGGCCACCATGGGATTCGTG |
| EGRev3 | CAGCTGTTATCCTGCCCTAGTAAT |
| XynFW3 | CAGCTGGCCACCATGGGTTTCGT |
| XynRev3 | CAGCTGTTAAGGTGTATTACCATATCC |
| EGFW4 | TGTCAGAAATTAGAGAGTTTATGGTTA |
| EGRev4 | TAATTGTAGTGGTAGGTGTCTGAGGTG |
| EGFW5 | GATACGGACCAGAAGGTTTTAAGGATA |
| XynFW4 | CTTCATCAAGTTCTTCAAGTTCTGGAG |
| XynRev3 | AAAGGGTTTGTAGTCCATCCATACAAT |
| RT-PCR/qRT-PCR-Xyn-FW | CAGACCTCCAGGAGGTCAAGGGGTCCAGTT |
| RT-PCR/qRT-PCR-Xyn-Rev | AGTACCTCCGGTCCTTCTCTGTTCGGCATGC |
| RT-PCR/qRT-PCR-EG-FW | GAATAGGATTGGTTCCATGGGCTCCGTCGGA |
| RT-PCR/qRT-PCR-EG-Rev | TATCTCCCCAATTATCTGCGAGCGTTGCGA |
